# Epigenetic Modifications as Novel Biomarkers for Diagnosis, Prognosis, and Therapeutic Targeting in Thyroid, Pancreas, and Lung Neuroendocrine Tumors

**DOI:** 10.3390/jcm14082622

**Published:** 2025-04-11

**Authors:** Federica Colapietra, Paola Della Monica, Raffaella Di Napoli, Fábio França Vieira e Silva, Giuliana Settembre, Maria Michela Marino, Andrea Ballini, Stefania Cantore, Marina Di Domenico

**Affiliations:** 1 Department of Precision Medicine, University of Campania Luigi Vanvitelli, Via Abramo Lincoln, 5, 81100 Caserta, Italy; federica.colapietra@unicampania.it (F.C.); fabio.francavieiraesilva@unicampania.it (F.F.V.e.S.); giuliana.settembre@policliniconapoli.it (G.S.); mariamichela.marino@unicampania.it (M.M.M.); andrea.ballini@unifg.it (A.B.); marina.didomenico@unicampania.it (M.D.D.); 2 Department of Advanced Medical and Surgical Science, University of Campania Luigi Vanvitelli, Via Abramo Lincoln, 5, 81100 Caserta, Italy; paola.dellamonica@unicampania.it; 3 Department of Experimental Medicine, University of Campania Luigi Vanvitelli, Via Abramo Lincoln, 5, 81100 Caserta, Italy; raffaella.dinapoli@unicampania.it; 4 Campania Regional Centre for Pharmacovigilance and Pharmacoepidemiology, 80138 Naples, Italy; 5 Department of Pathology, Health Research Institute of Santiago de Compostela (FIDIS), Santiago de Compostela University Clinical Hospital, University of Santiago de Compostela, Choupana Street, s/n, 15706 Santiago de Compostela, Spain; 6 Department of Clinical and Experimental Medicine, University of Foggia, Via Napoli, 20, 71122 Foggia, Italy; 7 Department of Life Science, Health and Health Professions, Link Campus University, 00165 Rome, Italy

**Keywords:** neuroendocrine neoplasms, well-differentiated neuroendocrine tumors, epigenetic alterations, DNA methylation

## Abstract

Neuroendocrine neoplasms (NENs) comprise a heterogeneous tumor group arising from neuroendocrine cells, commonly originating in the gastroenteropancreatic tract and bronchopulmonary system. Their incidence has risen significantly, owing to improved diagnostic techniques and increased clinical recognition. While previous reviews have explored the molecular and genetic basis of NENs, limited attention has been given to the role of epigenetic modifications, particularly DNA methylation, in tumorigenesis and disease progression. This review focuses on lung, pancreas, and thyroid well-differentiated neuroendocrine tumors (NETs), highlighting epigenetic mechanisms, particularly DNA methylation, as promising biomarkers for early diagnosis and risk stratification. Aberrant DNA methylation can silence key tumor suppressor genes, including RASSF1A and CDKN2A, thereby promoting tumorigenesis. Integrating DNA methylation profiles with conventional biomarkers such as chromogranin A (CgA) may enhance diagnostic accuracy and inform therapeutic strategies. Emerging epigenetic therapies offer potential avenues for personalized treatment based on molecular profiling. Unlike prior reviews that broadly cover genetic and epigenetic changes in NENs, this review uniquely emphasizes the translational potential of epigenetic biomarkers in clinical practice. By synthesizing recent findings and evaluating their clinical implications, we aim to bridge the gap between molecular research and practical applications in diagnosis, prognosis, and therapy.

## 1. Introduction

Neuroendocrine neoplasms (NENs) are a heterogeneous group of tumors arising from neuroendocrine cells in various organs. While the gastroenteropancreatic tract and bronchopulmonary system are the most common origin sites, these tumors can develop in almost any tissue and account for approximately 2% of all malignant tumor diagnoses in the United States [1,2]. Advances in diagnostic techniques and increased clinical recognition have led to a marked rise in their reported incidence and prevalence over the past few decades [3]. A study by Hallet et al. reported that NEN incidence increased 2.5-fold between 1994 and 2009, reaching an annual rate of 5.86 cases per 100,000 people in 2009 [4]. Clinically, NENs can present as functional tumors, causing hormone-related syndromes, or as nonfunctional tumors, which are frequently detected incidentally or at an advanced stage, often with liver metastases.

The NEN classification has undergone multiple revisions in recent years, with the 2022 World Health Organization (WHO) update distinguishing well-differentiated neuroendocrine tumors (NETs) from poorly differentiated neuroendocrine carcinomas (NECs), as they differ significantly in morphology, clinical behavior, and prognosis. NETs often exhibit an indolent course but may require long-term treatment, which can include somatostatin analogs (SSAs), targeted therapies, such as mechanistic target of rapamycin (mTOR) and Vascular Endothelial Growth Factor (VEGF) inhibitors, chemotherapy, and peptide receptor radionuclide therapy (PRRT) [2]. Delayed diagnosis remains a major challenge in NENs, frequently leading to liver metastasis detection at presentation. Prognosis varies widely depending on stage: patients with localized disease have a more favorable outlook, with 5-year survival rates ranging from 78% to 93%, whereas metastatic disease is associated with significantly lower 5-year survival rates (19–38%). However, recent advances in diagnostic accuracy and treatment efficacy have led to significant improvements in survival, in some cases enabling disease downstaging [5].

While the prior literature has extensively reviewed the genetic landscape and histopathological classification of NENs, a comprehensive synthesis of the role of epigenetic modifications—particularly DNA methylation patterns—as biomarkers for early diagnosis and prognosis remains lacking [6].

This review aims to fill this void by integrating recent findings on epigenetic alterations and their potential applications in clinical management.

**Figure 1:**
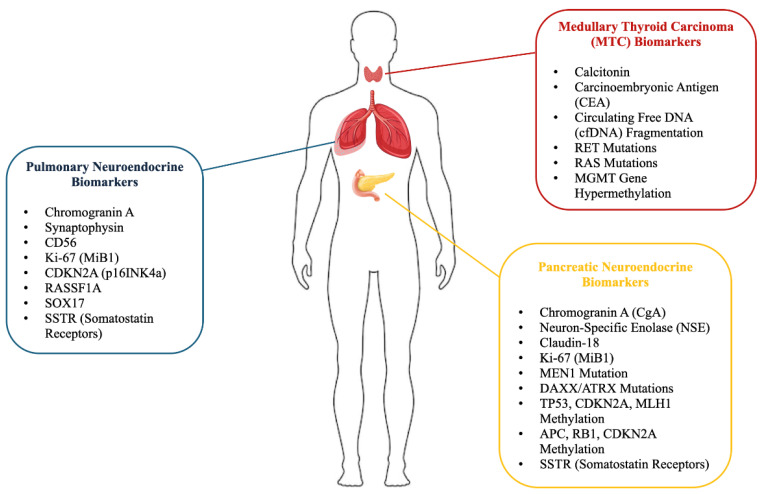
Biomarkers in pulmonary, medullary thyroid, and pancreatic neuroendocrine tumors. For pulmonary neuroendocrine tumors, biomarkers such as Chromogranin A, Synaptophysin, CD56, and Antigen Kiel 67 (Ki-67 or MiB1) are highlighted as important tumor presence and proliferation indicators. In medullary thyroid carcinoma (MTC), biomarkers including Calcitonin, Carcinoembryonic Antigen (CEA), circulating free DNA (cfDNA) fragmentation, and genetic mutations such as REarranged during Transfection (RET) and rat sarcoma (RAS) are essential for diagnosis and prognosis. Additionally, epigenetic markers like O6-methylguanine-DNA methyltransferase (MGMT) gene hypermethylation, cyclin-dependent kinase inhibitor 2A (CDKN2A or p16INK4a), Ras Association Domain Family Member 1 (RASSF1A), and SRY-related HMG-box 17 (SOX17) are shown to play significant roles. In pancreatic neuroendocrine tumors, chromogranin A (CgA), Neuron-Specific Enolase (NSE), Claudin-18, Ki-67 (MiB1), and genetic alterations such as Multiple Endocrine Neoplasia, Type 1 (MEN1) mutation, Death Domain Associated Protein (DAXX), and alpha-thalassemia mental retardation X-linked (ATRX) mutations, and methylation of genes like tumor protein p53 (TP53), cyclin-dependent kinase inhibitor 2A (CDKN2A), and MutL Homolog 1 (MLH1) are presented as critical biomarkers. The figure also includes the role of somatostatin receptors (SSTRs) in all three tumor types, further contributing to tumor diagnostics and therapeutic targeting.

Recent advances have fueled a growing interest in identifying molecular biomarkers and epigenetic mechanisms to enhance NEN diagnosis and management (Figure 1). Among these, DNA methylation has emerged as a promising diagnostic and prognostic marker, as well as a potential therapeutic target. Aberrant key gene methylation involved in cell proliferation and endocrine regulation may contribute to NEN tumorigenesis and progression, representing a rapidly evolving area of research [7].

This review builds upon prior studies by focusing on lung, pancreas, and thyroid well-differentiated NETs, analyzing the role of epigenetics—particularly DNA methylation—as a biomarker and its potential impact on early diagnosis, risk stratification, and therapeutic strategies. Additionally, recent advances in pharmacological treatments will be discussed, highlighting how personalized approaches based on molecular insights can significantly improve patient outcomes. By clarifying the mechanistic underpinnings and clinical relevance of epigenetic modifications, this review provides a novel contribution to the existing body of literature, helping to bridge gaps in knowledge and translate molecular discoveries into actionable clinical strategies. However, despite these advances, gaps remain in the literature regarding the specificity and sensitivity of epigenetic biomarkers for NETs. In particular, the clinical validation of these biomarkers is still limited, and larger studies are needed to determine their diagnostic and prognostic efficacy.

## 2. Epigenetic Modifications in Tumorigenesis

**Figure 2:**
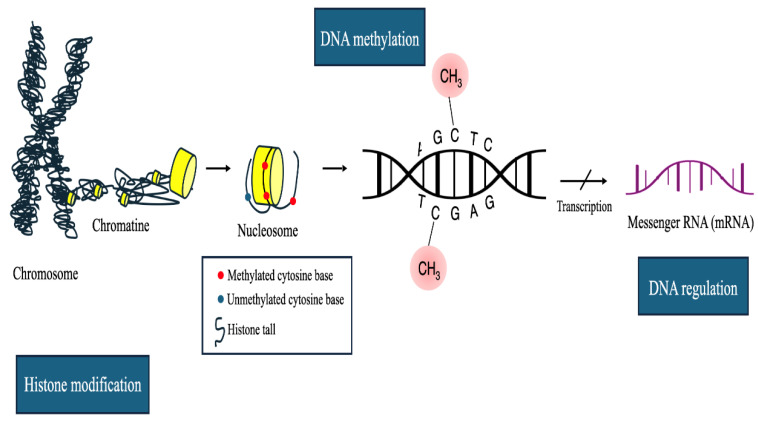
Epigenetic modifications in gene regulation. The chromatin structure, consisting of DNA wrapped around histone proteins to form nucleosomes, is depicted. DNA methylation is shown as the addition of methyl groups to cytosine bases (5-methylcytosine) within the cytosine–guanine (CpG) dinucleotides or CpG islands, with methylated (5mC) and unmethylated cytosine bases (C) marked. Histone modifications, such as methylation and acetylation of the histone tails, are represented as chemical marks that alter chromatin structure and influence gene accessibility. These modifications can either condense the chromatin, repressing transcription, or relax the chromatin, facilitating transcription. Transcription factors initiate the process of transcription, where messenger RNA (mRNA) is synthesized from the DNA template, enabling gene regulation expression by these epigenetic marks.

Epigenetic modifications are heritable changes in gene expression that do not involve alterations to the DNA nucleotide sequence [8]. The primary epigenetic mechanisms include DNA methylation, post-translational modifications of histones, and regulation by non-coding RNAs (Figure 2).

These processes are crucial for controlling DNA accessibility to transcription factors and regulating gene expression. Histone modifications, such as the addition of methyl, acetyl, or phosphate groups to specific amino acid residues in histone proteins, directly influence chromatin structure and gene transcription [9]. Similarly, microRNAs (miRNAs) and long non-coding RNAs (lncRNAs) contribute an additional layer of epigenetic regulation, modulating gene expression at the post-transcriptional level. Disruptions in these epigenetic mechanisms are commonly observed in tumors and are recognized as critical contributors to tumorigenesis. DNA methylation, the most well-known epigenetic marker, was first identified in human tumors as global hypomethylation, followed by the discovery of hypermethylated tumor suppressor genes, and more recently, miRNA gene inactivation through DNA methylation [10]. These findings have underscored the impact of epigenetic modifications on gene expression, spurring initiatives like the human genome projects and epigenetic therapy development. Moreover, it is now understood that DNA methylation occurs within a complex chromatin network, influenced by changes in histone structure, which is frequently altered in cancer cells.

### Methylation Pattern Relevance in NET Pathogenesis

Epigenetic mechanisms in lung NETs play a crucial role in tumorigenesis, influencing gene expression through DNA methylation and histone modifications, which may serve as potential biomarkers for diagnosis and therapeutic targets.

CpG islands, genome regions with a high cytosine–guanine dinucleotide density, are essential for regulating gene expression, especially when located in gene promoters. In NETs, these regions are often subjected to hypermethylation, an epigenetic modification that leads to the silencing of critical tumor suppressor genes. This modification alters chromatin structure, preventing transcription factors from accessing target genes and inhibiting their expression [11].

A prominent example is the Ras Association Domain Family Member 1A (RASSF1A) gene, whose promoter is frequently hypermethylated in NETs, especially in pancreatic and pulmonary NETs [12]. RASSF1A is crucial for regulating the cell cycle and inducing apoptosis, and its silencing through aberrant methylation results in its tumor-suppressive function loss. Similarly, the CDKN2A gene, which encodes the p16 protein responsible for inhibiting cyclin-dependent kinases and controlling cell cycle progression, is commonly hypermethylated in NETs, disrupting the regulation of cell proliferation.

In addition, chromogranin A (CgA), a biomarker widely used in clinical practice for diagnosing and monitoring NETs, is not directly linked to methylation but can be influenced by epigenetic modifications. These modifications affect neuroendocrine regulatory processes and may alter CgA expression. Combining DNA methylation profiles with CgA levels represents a promising strategy to enhance diagnostic and prognostic sensitivity, providing a more comprehensive understanding of the molecular mechanisms underlying NETs [13]. Elevated CgA levels are found in 90% of gastrointestinal NETs, with a sensitivity of 57–63.3% and specificity of 55.6–71.4% in distinguishing patients with metastases [14].

The CpG island’s methylation role, along with complementary biomarkers like CgA, highlights its potential in guiding early diagnosis, disease monitoring, and the development of targeted therapeutic strategies for NETs [11].

Another widely used biomarker is Neuron-Specific Enolase (NSE), which is often assessed alongside CgA. While NSE reflects tumor activity, its specificity is lower than that of CgA, as elevated NSE levels can also be observed in other malignancies, such as lung cancer. Nevertheless, NSE offers additional diagnostic value when used in conjunction with other markers [15,16]. In pancreatic NETs, the sensitivity of NSE varies between 30% and 50%, while in gastroenteropancreatic NETs (GEP-NETs) a sensitivity of 38% has been reported, which is lower than that of CgA (59%). However, the specificity of NSE is relatively high at 73% and has been shown to be higher than that of CgA in some clinical contexts, suggesting its possible complementary use in the diagnosis and prognostic stratification of NETs [17].

Claudin 18 has recently emerged as a promising biomarker for both pancreatic cancer and lung adenocarcinoma [18]. Its expression has been linked to favorable immune responses and may assist in patient stratification based on prognosis. Although its role in endocrine pancreatic tumors is still under investigation, early studies suggest that Claudin 18 may improve diagnostic and therapeutic approaches [19].

## 3. Lung Neuroendocrine Tumors

Lung NETs are a heterogeneous neoplasm group that originates from neuroendocrine cells within the respiratory epithelium. They are classified into four main categories: typical carcinoid, atypical carcinoid, large-cell neuroendocrine carcinoma (LCNEC), and small-cell lung carcinoma (SCLC). Typical and atypical carcinoids are generally well-differentiated tumors with low to moderate proliferative rates, while LCNEC and SCLC are poorly differentiated with high proliferative activity and aggressive clinical behavior [20].

Typical carcinoids are associated with a relatively favorable prognosis, often being localized at diagnosis time, and typically show indolent growth. In contrast, atypical carcinoids have a higher propensity for metastasis, particularly to regional lymph nodes and distant organs. LCNEC and SCLC are more aggressive and typically present with advanced-stage disease at diagnosis. These subtypes are associated with poor prognosis due to their rapid growth and tendency for early metastasis. Recent advances in diagnostic techniques, including the use of immunohistochemical (IHC) markers such as chromogranin A, synaptophysin, and CD56, have significantly enhanced the ability to differentiate and classify lung NETs [21,22].

### 3.1. Epigenetic Modifications in Pulmonary Neuroendocrine Tumors

In PanNETs (pancreatic neuroendocrine tumors), aberrant DNA methylation and histone modifications contribute to tumor progression, affecting key regulatory pathways and highlighting the potential of epigenetic biomarkers for risk stratification and personalized treatment [10]. Aberrant methylation of promoter regions in tumor suppressor genes often leads to their silencing, facilitating tumor growth and progression.

A key example is CDKN2A gene hypermethylation, which encodes the p16INK4a protein (Table 1).

This modification, frequently observed in non-small-cell lung cancer (NSCLC), disrupts essential cell cycle control pathways [11]. Other genes, such as RASSF1A, are also commonly methylated in lung endocrine tumors. RASSF1A methylation induces genomic instability, a hallmark of cancer progression [23,24]. Additionally, SOX17 methylation impairs cellular regulation and contributes to tumorigenesis [25,26].

These gene methylations, including CDKN2A, can influence tumor behavior and therapeutic response [27], making them promising candidates for early diagnosis and therapeutic intervention. Ongoing research continues to explore the clinical potential of DNA methylation as a diagnostic and prognostic tool in pulmonary NETs.

### 3.2. Pulmonary Neuroendocrine Tumor Diagnosis

Pulmonary NET diagnosis relies on a combination of imaging techniques, endoscopic procedures, and histopathological analysis to identify the tumor, assess its extent, and classify its histological subtype. The primary diagnostic modalities include the following.

#### 3.2.1. Computed Tomography (CT)

CT provides detailed information about the tumor’s location, size, and margins. Typical carcinoids (≤2 cm) usually present as nodules with smooth margins, while atypical carcinoids (≥4 cm) have irregular margins. CT is also helpful in detecting secondary complications such as lobar atelectasis, obstructive pneumonia, or calcifications, which are present in approximately 30% of carcinoids. Enhanced contrast imaging reveals marked tumor stroma vascularity. In SCLC, CT may show a central mass with mediastinal lymph node involvement in 90–95% of cases [20,28].

#### 3.2.2. Chest X-Ray

Chest X-ray is useful first-line investigation, but less sensitive than CT for detecting smaller lesions or lymph node changes [29].

#### 3.2.3. PET-CT (Positron Emission Tomography)

PET-CT is critical for assessing tumor location, size, local invasion, and staging metastases. Fluorodeoxyglucose (FDG) PET distinguishes tumors with high metabolic activity (e.g., atypical carcinoids, LCNEC, and SCLC) from those with low metabolic activity (e.g., typical carcinoids). However, FDG PET sensitivity is limited in CT scans. Somatostatin receptor-specific tracers (such as 68Ga-DOTATATE or 68Ga-DOTATOC) are preferred for detecting well-differentiated tumors [20].

#### 3.2.4. Octreotide Scintigraphy and Somatostatin Receptor Imaging

Somatostatin receptors (SSTRs), which are expressed in 80–90% of NETs, can be visualized using scintigraphy or PET with radiolabeled analogs. Undifferentiated tumors tend to express SSTRs at lower frequencies. While IHC is less sensitive than scintigraphy, it can confirm SSTR expression, which is an important therapeutic target for SSAs (e.g., octreotide and lanreotide) [20,28].

#### 3.2.5. Bronchoscopy, Endosonography, and Biopsy

Bronchoscopy allows direct endobronchial lesion visualization, while needle aspiration-guided endosonography (EBUS) aids in sampling mediastinal lymph nodes. However, biopsies in highly vascularized carcinoids carry a significant risk of bleeding [29].

#### 3.2.6. Histopathological Analysis of Biopsy Specimens

Histopathological examination is essential to confirm the diagnosis and classify the tumor subtype. IHC markers such as chromogranin A, synaptophysin, and CD56 are used to confirm the neuroendocrine neoplasm nature. The Ki-67 proliferation index (MiB1) is a key parameter for differentiating low-grade carcinoids (typical and atypical) from high-grade neuroendocrine tumors (LCNEC and SCLC) [30]. In some cases, small biopsies or cytology may not provide sufficient material for a definitive diagnosis. For example, the LCNEC histological features resemble NSCLC and SCLC, making diagnosis challenging. Specific clinicopathological and molecular features are required to distinguish LCNEC from these other lung cancer types [31,32]. A Phase II clinical trial found that 27.5% of patients initially diagnosed with LCNEC were later reclassified, primarily to SCLC. Another study revealed that only 53% of patients were correctly diagnosed with LCNEC at initial presentation, with the remaining 47% misclassified as NSCLC [33,34].

### 3.3. Pulmonary Neuroendocrine Tumor Therapies

NETs require targeted therapeutic strategies due to their distinct molecular characteristics. Chemotherapy remains the main treatment option, especially for advanced or aggressive forms. In SCLC, cisplatin and etoposide (EP)-based regimens are the standard decision, demonstrating high efficacy in slowing disease progression and improving survival. Similarly, treatment for LCNEC largely relies on data extrapolated from SCLC trials, with cisplatin as the primary agent. Retrospective studies have shown variable objective response rates (ORRs), with some reports suggesting up to 50% response to cisplatin-based regimens [35]. However, alternative combinations, such as irinotecan and platinum, have not demonstrated superior benefits over the EP regimen.

Adjuvant chemotherapy with the EP regimen is particularly recommended for patients with stage IB to IIIA localized LCNEC after complete resection (R0), as it has been shown to improve overall survival compared to other treatments. Specifically, studies indicate that adjuvant chemotherapy for stage IB LCNEC is associated with a significant increase in overall survival, with a hazard ratio of 0.67, reflecting a 33% reduction in the risk of death compared to those who did not receive adjuvant therapy [33,36].

Carboplatin, an alternative for patients who are intolerant to cisplatin, is limited by the lack of robust prospective data supporting its efficacy in this setting [37]. For advanced LCNEC, treatment options are expanding to include radiotherapy and innovative therapies such as radioembolization with labeled somatostatin analogs, which can improve the quality of life for patients who are not candidates for surgery [38].

Current evidence emphasizes the need for personalized treatment strategies based on LCNEC molecular characteristics, and highlights the necessity for more comprehensive clinical trials to establish standardized treatment protocols [39].

Emerging targeted therapies are increasingly being investigated for pulmonary NETs. Somatostatin receptor-targeted peptide receptor radionuclide therapy (PRRT) using 177Lu-DOTATATE has demonstrated promising results in well-differentiated lung NETs expressing somatostatin receptors [40].

Studies have reported improved progression-free survival (PFS) and symptomatic relief in patients treated with PRRT. Additionally, immunotherapy approaches such as immune checkpoint inhibitors targeting the programmed cell death protein 1 and its ligand (PD-1/PD-L1) pathways are being explored in poorly differentiated subtypes like SCLC and LCNEC, although their efficacy remains variable and requires further investigation [41,42,43].

Molecular profiling has paved the way for personalized therapies targeting specific genetic alterations. Delta-like ligand 3 (DLL3) inhibitors like rovalpituzumab tesirine have shown potential in preclinical studies targeting DLL3 expression in SCLC but requires further validation in clinical trials [44].

mTOR inhibitors, like everolimus, have demonstrated activity in extrapulmonary NETs and are being evaluated for lung NETs due to their role in inhibiting tumor cell proliferation and angiogenesis [45,46]. VEGF inhibitors, like bevacizumab, have been studied in combination with chemotherapy regimens for advanced lung NETs but require more extensive clinical data [47,48].

In summary, advances in understanding lung NETs’ molecular and genetic basis have facilitated the development of increasingly targeted and personalized therapeutic approaches. The integration of chemotherapy, radiotherapy, and other pharmacological strategies is driving significant improvements in patients’ clinical management and outcomes.

## 4. Pancreatic Neuroendocrine Tumors

Pancreatic neuroendocrine tumors (PanNETs) are a group of rare neoplasms that arise from the neuroendocrine cells of the pancreas [49].

According to the 2017 and 2019 WHO classifications of digestive system tumors, pancreatic neuroendocrine neoplasms (pNENs) are categorized based on morphology into two main groups: pancreatic neuroendocrine carcinoma (pNEC), which demonstrates clear neuroendocrine lineage, and well-differentiated pancreatic neuroendocrine tumors (WD-pNETs), formerly referred to as “islet cell tumors” [50].

Clinically, pNENs are further divided into functional and nonfunctional tumors. Functional pNENs are characterized by the ability to secrete hormones or peptides, leading to clinical syndromes caused by hormone overproduction (e.g., insulinomas). Nonfunctional pNENs, on the other hand, do not cause a hormone-related syndrome but may secrete detectable peptide hormones, usually identifiable through biopsy [51].

Most pNENs are nonfunctional, while a low percentage are functioning tumors [52,53]. This distinction is important for clinical presentation, diagnosis, and treatment strategies. Functional pNENs include insulinomas, gastrinomas, glucagonomas, vasoactive intestinal peptide tumors (VIPomas), somatostatinomas, growth hormone-releasing hormone (GHRH)-secreting tumors, and, in very rare cases, tumors that secrete luteinizing hormone, renin, insulin-like growth factor 2, or erythropoietin [38]. Among the functioning pNENs, insulinomas are the most common, followed by gastrinomas, while VIPomas and glucagonomas are much rarer [54].

Molecular subtyping is critical for selecting the appropriate therapeutic approach in many tumor types, and this concept has recently been applied to pNEN evaluation. By using multi-omics analyses, researchers have been able to classify pNENs into subtypes with distinct characteristics, which may inform treatment decisions.

In recent years, genetic and epigenetic research has become increasingly important in understanding the pathogenic mechanisms underlying pancreatic neuroendocrine neoplasms.

Recent studies suggest that microRNAs can influence metabolic pathways in cancer cells. For example, miR-423-5p has been shown to modulate glucose and amino acid metabolism in prostate cancer cells, highlighting a potential role in tumor metabolic reprogramming [55]. Epigenetic alterations, such as DNA methylation, play a significant role in regulating gene expression and contribute to malignant transformation in pNENs [56].

### 4.1. Epigenetic Modifications in Pancreatic Neuroendocrine Tumors

PanNETs are associated with mutations in several key genes, including MEN1, DAXX, ATRX, and TP53, which are involved in critical processes such as cell growth regulation, DNA repair, and genomic stability maintenance [57] (Table 1). Among these, MEN1 is the most commonly mutated gene in sporadic PanNETs and is also a major factor in multiple endocrine neoplasia type 1, a genetic disorder that increases the risk of neuroendocrine tumors across multiple organs, including the pancreas. The MEN1 mutation results in the loss of menin protein function, a key regulator of gene transcription and genomic stability. This loss leads to uncontrolled cell proliferation and disrupts tumor growth regulation, contributing to both sporadic and familial PanNETs [58].

Mutations in the DAXX and ATRX genes are also important. These genes play roles in chromatin structure regulation and telomere protection. Alterations in DAXX and ATRX often lead to genomic instability, which contributes to increased tumor aggression and a poorer prognosis in PanNET patients [59].

In addition to genetic mutations, epigenetic modifications play a crucial role in tumor initiation and progression. Studies have shown that in PanNETs, the methylation of key genes such as TP53, CDKN2A, and MLH1 is often dysregulated [60,61]. These genes are essential for cell cycle control and DNA repair, and their abnormal methylation impairs the cell’s ability to respond to DNA damage, increasing the risk of neoplastic transformation. Methylation of MEN1 has also been observed in some PanNETs, suggesting that epigenetic changes may work together with genetic mutations to drive tumor development [62,63].

Alterations in methylation patterns can serve as important predictors of tumor aggressiveness and treatment response, providing valuable prognostic insights. For example, abnormal methylation of genes like APC and MGMT is associated with a higher risk of metastasis and poor overall survival [64]. Similarly, methylation changes in APC, RB1, and CDKN2A have been linked to an increased risk of metastasis and worse prognosis [65]. These methylation patterns have potential as biomarkers for early diagnosis and monitoring of tumor progression.

Interventions aimed at modifying methylation patterns, such as DNA methyltransferase inhibitors, are being explored as potential therapeutic strategies for neuroendocrine tumors [66]. Additionally, the tumor microenvironment, including factors such as hypoxia, inflammation, and stromal dysfunction, can influence epigenetic changes. These factors may trigger alterations in DNA methylation and histone modifications, further impacting gene expression and tumor progression [67]. In conclusion, epigenetic alterations, including global DNA hypomethylation and histone modifications, are implicated in the pathogenesis of thyroid NETs, highlighting their importance in disease progression and their potential as novel therapeutic targets.

### 4.2. Pancreatic Neuroendocrine Tumor Diagnosis

PanNETs diagnosis involves a multimodal approach, combining imaging, histological analysis, and biomarkers. Imaging techniques, including CT, magnetic resonance imaging (MRI), and PET with specific tracers like gallium-68-DOTATATE, are crucial for assessing the tumor’s location and extent [68] (Table 2).

Histopathology remains the gold standard for confirming the diagnosis, with the Ki-67 index being used to determine tumor grade [69]. Serum biomarkers, such as chromogranin A and hormones specific to functioning tumors, further aid in diagnosis and monitoring disease progression [70].

### 4.3. Pancreatic Neuroendocrine Tumor Therapies

Treatment for PanNETs depends on the tumor’s grade and stage. Surgical intervention remains a viable option for small, resectable tumors; however, metastatic and locally advanced pancreatic neuroendocrine tumors (pNETs) present considerable mortality risks, with treatment efficacy historically being limited [71]. Previously, streptozocin-based therapies were the primary treatment modality, but recent advancements have expanded the therapeutic arsenal available for pNET management.

Contemporary chemotherapeutic options now include agents such as temozolomide, somatostatin analogs (SSAs), and targeted therapies like everolimus and sunitinib [72,73] (Table 3). Ongoing clinical trials are exploring various combination regimens that incorporate these newer agents alongside targeted therapies, including pazopanib.

Cytoreductive surgery and somatostatin analogs are crucial in the management of these tumors, while palliative care strategies—such as molecular targeted therapies, peptide receptor radionuclide therapy, and chemotherapy—are typically reserved for patients who do not respond to somatostatin analogs [72].

A deeper understanding of the genetic underpinnings of pancreatic neuroendocrine tumors and the exploration of immunotherapy may pave the way for promising future research avenues (R1).

For more advanced stages, other medications, such as everolimus (an mTOR inhibitor) and sunitinib (a tyrosine kinase inhibitor), are used. Both have shown proven efficacy in extending progression-free survival [74].

Emerging therapeutic strategies include epigenetic therapies, such as DNA methyltransferase and histone deacetylase inhibitors, which may help restore the expression of silenced tumor suppressor genes. Another promising approach is PRRT using lutetium-177, which has demonstrated encouraging results in controlling tumor growth in patients with advanced disease [75].

## 5. Thyroid Neuroendocrine Tumors

Thyroid NETs are a rare and heterogeneous neoplasm group originating from neuroendocrine cells within the thyroid gland. These cells, known as parafollicular or C cells, are primarily responsible for producing calcitonin, a hormone essential for regulating blood calcium levels [76]. The tumor is classified as neuroendocrine due to the shared characteristics between C cells and other neuroendocrine cells, including the ability to synthesize hormone peptides and express specific markers [77].

Neuroendocrine tumors are distinguished by their unique cellular origin, complex biological behavior, and clinical features, which can vary widely between patients. In the endocrine and neuroendocrine tumor 2022 WHO classification, medullary thyroid carcinoma (MTC) was classified as one of the major histologic types of thyroid tumors, accounting for about 5–10% of all thyroid malignancies [78]. The new classification system is based on the cell of origin and clinical risk, with major categories including follicular cell-derived neoplasms (FDNs) and parafollicular cell (C cell)-derived tumors, such as malignant MTC.

MTC is characterized by rapid growth and a high propensity for metastasis, which presents significant challenges for early detection and treatment. Molecular genetic research has provided valuable insights into MTC pathogenesis and identified potential therapeutic targets. The disease occurs in hereditary and sporadic forms, with the hereditary variant associated with multiple endocrine neoplasia type 2 (MEN2) syndromes. MEN2 encompasses three subtypes: type 2A (MEN2A), type 2B (MEN2B), and familial medullary thyroid carcinoma (FMTC), each associated with specific clinical features and germline RET mutations [79]. Managing MTC requires a multidisciplinary approach involving endocrinology, genetics, and oncology. In MTC, DNA methylation patterns influence tumor behavior, with MGMT gene hypermethylation emerging as a potential diagnostic marker. Additionally, cfDNA methylation signatures could serve as non-invasive tools for disease monitoring [80].

Recent research has highlighted the molecular mechanisms underlying MTC, with RET and RAS mutations identified as key contributors to its pathogenesis. RET mutations are associated with a more aggressive phenotype, characterized by a higher lymph node involvement risk, increased distant metastasis likelihood, and a poorer overall prognosis compared to MTC cases with other mutations [81]. RAS mutations, found in approximately 30% of MTC cases, are common in various cancers and are significant because they can disrupt normal RAS protein regulation, potentially leading to unregulated cell proliferation and tumorigenesis [82,83].

Epigenetic mechanisms also play an important role in MTC progression, with pathways distinct from those linked to RET and RAS mutations. A study analyzing global DNA methylation in peripheral blood leukocytes from individuals with MTC revealed increased methylation levels, which may be influenced by environmental factors rather than the germline mutations typically seen in hereditary MTC.

### 5.1. Epigenetic Modifications in Thyroid Neuroendocrine Tumors

Recent studies have underscored methylation’s role in MTC, revealing distinct patterns that could enhance diagnostic precision. For instance, circulating cell-free DNA (cfDNA) from MTC patients has been shown to exhibit specific methylation signatures [84]. These signatures may complement traditional diagnostic markers like serum calcitonin, which can sometimes provide ambiguous results due to non-specific elevations. The ability to detect methylation changes in cfDNA marks a significant advance in developing non-invasive diagnostic tools [85].

Interestingly, methylation changes have also been linked to patient outcomes. Research on intestinal NETs has shown that hypomethylation of genes involved in G-protein-coupled receptor (GPCR) signaling pathways correlates with poorer survival rates. While this finding pertains to NETs outside the thyroid, it highlights the broader relevance of methylation in NETs, suggesting that similar mechanisms may also be at play in MTC [86].

For specific DNA regions, methylation has become a key area of research in MTC. Notably, hypermethylation of the CG_16698623 dinucleotide in the MGMT gene has been identified as a potential diagnostic marker. This epigenetic alteration is significantly more prevalent in MTC tissues than healthy controls, indicating its potential for distinguishing malignant from benign conditions [87] (Table 1).

The fragmentation patterns of circulating cfDNA have garnered attention as a diagnostic tool. Studies have shown that patients with MTC exhibit a higher proportion of short DNA fragments (short fragment fraction, SFF) in their plasma compared to healthy individuals or those in remission. Analyzing cfDNA fragmentation offers a minimally invasive approach to detect MTC and monitor disease progression or recurrence, potentially serving as a liquid biopsy technique [84].

cfDNA, released into the bloodstream by various cells, has emerged as a valuable source for identifying diagnostic and prognostic biomarkers, including tumor-specific mutations and epigenetic alterations. Previous studies have primarily focused on detecting tumor-specific mutations in cfDNA to monitor minimal residual disease and treatment responses in MTC [88].

Calcitonin remains the cornerstone biomarker for diagnosing and monitoring MTC. Elevated serum calcitonin levels are highly suggestive of MTC; however, they lack absolute specificity, as other conditions such as chronic kidney disease or autoimmune thyroiditis can also elevate calcitonin levels. Sole reliance on calcitonin can lead to false positives or negatives, highlighting the need for additional biomarkers to improve diagnostic accuracy [89].

Exploring alternative circulating biomarkers, such as carcinoembryonic antigen (CEA) and Ca 19-9, has shown potential prognostic value in advanced MTC cases. These markers may help stratify patients based on disease severity and guide therapeutic decisions. While not specific to MTC, they could complement other diagnostic tests, particularly in cases where calcitonin levels are inconclusive [90].

Integrating emerging biomarkers like DNA methylation and cfDNA fragmentation into clinical practice could revolutionize MTC diagnosis and management. Combining these molecular tools with traditional markers like calcitonin and CEA may enable earlier detection, better risk stratification, and more personalized treatment strategies. Advances in molecular diagnostics have opened new avenues for identifying MTC with greater precision. Among the most promising developments are DNA methylation patterns (e.g., MGMT hypermethylation) and cfDNA fragmentation analysis, which offer non-invasive and highly specific diagnostic options. These biomarkers hold significant potential to complement traditional tools like calcitonin measurement, paving the way for improved patient outcomes. Further validation through large-scale studies is essential to establish their clinical utility and integration into routine care.

### 5.2. Thyroid Neuroendocrine Tumor Diagnosis

MTC diagnosis relies on integrating clinical, biochemical, and histopathological data. Parafollicular cells in the thyroid produce calcitonin, the primary biomarker for MTC diagnosis. Management typically includes thyroidectomy followed by central and/or lateral neck dissection. Postoperative monitoring involves clinical assessments, neck ultrasounds, and calcitonin level measurements. Recent studies have explored additional serum markers, with procalcitonin emerging as a promising candidate [91,92] (Table 2). In cases of persistence or recurrence, chemotherapy or multikinase inhibitors are used. The therapeutic landscape for advanced disease has notably expanded with RET-specific inhibitors, and future studies should evaluate potentially combining therapies.

Fine-needle aspiration (FNA) biopsy plays a crucial role in identifying distinct cytological features, offering insights into the tumor’s morphology and biological behavior. This diagnostic approach is especially valuable for distinguishing MTC from other thyroid neoplasms, combining cytological findings with biochemical markers and molecular analysis [93,94]. Additionally, FNA is a minimally invasive procedure typically well tolerated by patients, providing high-quality material for histological and genetic analysis. IHC aids diagnosis by detecting neuroendocrine markers, including chromogranin A, synaptophysin, and calcitonin, which help identify the NET nature and differentiate it from other thyroid pathologies. IHC also facilitates the evaluation of markers that assess tumor aggressiveness and identify potential therapeutic targets [95].

Genetic testing is essential for detecting RET mutations in hereditary cases. These tests confirm the diagnosis and help to identify asymptomatic carriers within at-risk families [96]. Early diagnosis in these individuals allows for preventive measures, such as prophylactic thyroidectomy, which can prevent MTC development. Furthermore, identifying specific mutations can guide targeted therapies, including tyrosine kinase inhibitors [97].

### 5.3. Thyroid Neuroendocrine Tumor Therapies

MTC management primarily involves surgery, the first-line treatment decision for curative intention. Total thyroidectomy, often combined with cervical lymph node dissection, is considered the standard approach to ensure complete excision of the tumor and any regional metastases, thereby minimizing local recurrence risk [98]. In advanced or metastatic cases, novel systemic therapies are necessary. Notably, RET-specific tyrosine kinase inhibitors like vandetanib and cabozantinib inhibit cellular signaling pathways critical for tumor proliferation and survival [99] (Table 3). These drugs, approved for treating metastatic or unresectable bone marrow malignancies, have significantly extended progression-free survival and improved patients’ quality of life. Additionally, recent research has investigated new tyrosine kinase inhibitors that show better toxicity profiles and greater specificity for the involved genetic mutations [100]. Targeted therapies such as tyrosine kinase inhibitors (TKIs) like lenvatinib and sorafenib have been approved for progressive, radioactive iodine-refractory metastatic DTC. The combination of B-Rapidly Accelerated Fibrosarcoma (BRAF) and mitogen-activated protein kinase kinase (MEK) inhibitors is also being explored for BRAF V600E-mutated anaplastic thyroid carcinoma [101,102].

## 6. Strengths and Limitations

This review provides a comprehensive and up-to-date overview of the current knowledge of epigenetic mechanisms involved in NETs, including recent studies and key findings in the field. The analysis of epigenetic alterations is addressed for different types of NETs (lung, pancreatic, and thyroid), highlighting similarities and differences in molecular mechanisms and clinical implications. This review discusses the potential use of epigenetic biomarkers for early diagnosis and risk stratification, as well as the potential use of epigenetic therapies in NETs.

Despite the promising potential of epigenetic biomarkers for early diagnosis and precision medicine in NETs, several challenges hinder their translation from bench to bedside. First, the reproducibility of epigenetic assays remains a major issue, as different methodologies often yield variable results. Second, standardization across laboratories is lacking, making it difficult to establish universal cut-off values for clinical application. Additionally, regulatory and ethical barriers must be addressed before integrating epigenetic biomarkers into routine diagnostics. Overcoming these hurdles will require large-scale, multicenter validation studies and the development of cost-effective, high-throughput technologies.

While epigenetic biomarkers offer significant promise, current findings have limitations. Most studies are retrospective, with small sample sizes and a lack of large-scale validation. Additionally, the interaction between epigenetic modifications and tumor microenvironment factors remains poorly understood. Addressing these gaps will require collaborative efforts among researchers, clinicians, and industry stakeholders to refine detection methodologies, develop clinically relevant animal and cell-based models, and integrate multi-omics approaches for a more comprehensive understanding of NET epigenetics.

## 7. Conclusions

Epigenetic modifications play a central role in tumor development and progression. Abnormal methylation patterns, such as those affecting key tumor suppressor genes, contribute to critical regulatory pathway silencing and driving tumorigenesis. Combining DNA methylation profiles with traditional biomarkers, like chromogranin A, holds promise for enhancing diagnostic accuracy and prognostic stratification. This integrated approach could lead to earlier detection, better monitoring of disease progression, and more personalized treatment plans. As research deepens into the large-scale epigenetic mechanisms of NENs, the potential for developing innovative diagnostic tools and targeted therapies grows. Ultimately, this progress will significantly improve patient outcomes and quality of life.

## Figures and Tables

**Table 1:** Biomarkers for pulmonary neuroendocrine tumors, pancreatic neuroendocrine tumors, and medullary thyroid carcinoma. NETs = neuroendocrine tumors; LCNEC = large-cell neuroendocrine carcinoma; SCLC = small-cell lung carcinoma; PanNETs = pancreatic neuroendocrine tumors; MTC = medullary thyroid carcinoma; SSTRs = somatostatin receptors; chromogranin A = CgA; NSE = Neuron-Specific Enolase; CEA = Carcinoembryonic Antigen; cfDNA = circulating free DNA; SFF = short fragment fraction.

NET Type	Biomarker	Tumor Subtype	Role	Clinical Significance
Pulmonary Neuroendocrine Tumors	Chromogranin A	All pulmonary NETs	Diagnostic	General marker for NETs, useful for disease monitoring
	Synaptophysin	All pulmonary NETs	Diagnostic	Confirms neuroendocrine nature
	CD56	All pulmonary NETs	Diagnostic	Commonly expressed in pulmonary NETs
	Ki-67 (MiB1)	All pulmonary NETs	Prognostic	Differentiates carcinoids (low index) from high-grade NETs (high index)
	CDKN2A (p16INK4a)	LCNEC, SCLC	Prognostic	Methylation promotes tumor progression
	RASSF1A	All pulmonary NETs	Prognostic	Methylation induces genomic instability
	SOX17	All pulmonary NETs	Prognostic	Silencing promotes tumorigenesis
	SSTRs	Typical and atypical carcinoids	Diagnostic and therapeutic	Target for somatostatin analog therapy
PanNETs	CgA	All PanNETs	Diagnostic	General marker for PanNETs, useful for disease monitoring
	NSE	All PanNETs	Diagnostic	Reflects tumor activity, but with lower specificity than CgA
	Claudin-18	All PanNETs	Prognostic	Associated with immune response and patient stratification
	Ki-67 (MiB1)	All PanNETs	Prognostic	Differentiates well-differentiated (low index) from high-grade tumors (high index)
	MEN1 Mutation	Sporadic and hereditary PanNETs	Genetic	Loss of function promotes tumor proliferation
	DAXX/ATRX Mutations	All PanNETs	Prognostic	Associated with chromatin remodeling and poor prognosis
	TP53, CDKN2A, MLH1 Methylation	All PanNETs	Epigenetic	Impairs DNA repair and tumor suppression, promoting malignancy
	APC, RB1, CDKN2A Methylation	All PanNETs	Prognostic	Linked to metastasis and reduced survival
	SSTRs	Well-differentiated PanNETs	Diagnostic and therapeutic	Target for somatostatin analog therapy and PRRT
MTC	Calcitonin	Peptide hormone	Primary diagnostic marker	Elevated in MTC, but also in renal failure and autoimmune thyroiditis
	CEA	Glycoprotein	Prognostic marker	Higher levels indicate advanced disease and poor prognosis
	cfDNA Fragmentation	Epigenetic marker	Non-invasive diagnostic tool	Increased SFF in MTC patients
	RET Mutations	Genetic alteration	Key molecular marker	Associated with hereditary and sporadic MTC, more aggressive phenotype
	RAS Mutations	Genetic alteration	Somatic mutation	Found in ~30% of MTC cases, linked to unregulated cell proliferation
	MGMT Gene Hypermethylation	Epigenetic marker	Potential diagnostic marker	More prevalent in MTC tissues than in healthy controls

**Table 2:** Diagnostic methods for pulmonary neuroendocrine tumors, pancreatic neuroendocrine tumors, and medullary thyroid carcinoma. CT = Computed Tomography; PET-CT = Positron Emission Tomography; FDG = Fluorodeoxyglucose; IHC = Immunohistochemistry; MRI = magnetic resonance imaging; NETs = neuroendocrine tumors; PanNETs = pancreatic neuroendocrine tumors; MTC = medullary thyroid carcinoma; EUS = Endoscopic Ultrasound; CEA = Carcinoembryonic Antigen; FNA = fine-needle aspiration; SRI = Somatostatin Receptor Imaging; SSTRs = somatostatin receptors.

NET Type	Diagnostic Modality	Utility	Characteristics
Pulmonary Neuroendocrine Tumors	CT	Localization and staging	Highlights margins, size, and associated complications
	Chest X-ray	Initial screening	Sensitive for large lesions
	PET-CT with FDG	Metabolic assessment	Differentiates tumors with low and high metabolic activity
	PET-CT with 68Ga-DOTATATE	Diagnosis of well-differentiated NETs	Highlights somatostatin receptors
	Octreotide Scintigraphy	Confirms SSTRs expression	Alternative to PET for assessing somatostatin receptors
	Bronchoscopy with Biopsy	Histological confirmation	Required for histopathological and immunohistochemical analysis
	IHC	Histological classification	Uses neuroendocrine markers (Chromogranin A, Synaptophysin, CD56)
	Molecular Analysis	Identification of genetic alterations	Detects mutations and epigenetic modifications for targeted therapies
PanNETs	CT	Localization and staging	Assesses tumor size, margins, and complications
	MRI	Staging	Superior contrast for soft tissues, useful for liver metastases
	PET-CT with 68Ga-DOTATATE	Diagnosis of well-differentiated PanNETs	Highlights somatostatin receptor expression
	FDG-PET	High-grade PanNETs	Identifies tumors with high metabolic activity
	EUS with Biopsy	Histological confirmation	Essential for grading and immunohistochemical analysis
	IHC	Tumor classification	Uses neuroendocrine markers (CgA, NSE, Claudin-18)
	Molecular Analysis	Identification of genetic alterations	Detects mutations and epigenetic changes for targeted therapies
MTC	Serum Calcitonin	Primary biomarker for MTC detection	Highly sensitive; elevated levels suggest MTC
	CEA	Prognostic marker	Correlates with tumor burden and progression
	FNA Cytology	Initial tissue diagnosis	May be inconclusive; requires calcitonin staining
	RET Proto-Oncogene Testing	Genetic screening	Identifies hereditary MTC in MEN2 syndromes
	Ultrasound (Neck)	First-line imaging for thyroid nodules	Detects suspicious nodules and lymphadenopathy
	CT/MRI	Staging and metastasis evaluation	Useful for advanced disease and lymph node assessment
	18F-FDG PET/CT	Detects metastatic or recurrent disease	Useful in cases with negative conventional imaging
	SRI	Functional imaging	Evaluates tumor expression of somatostatin receptors

**Table 3:** Treatment options for pulmonary neuroendocrine tumors, pancreatic neuroendocrine tumors, and medullary thyroid carcinoma. NETs = neuroendocrine tumors; LCNEC = large-cell neuroendocrine carcinoma; SCLC = small-cell lung carcinoma; PanNETs = pancreatic neuroendocrine tumors; MTC = medullary thyroid carcinoma; tyrosine kinase inhibitors = TKIs; peptide receptor radionuclide therapy = PRRT.

NET Type	Therapy	Indication	Effect
Pulmonary Neuroendocrine Tumors	Chemotherapy (EP: Cisplatin + Etoposide)	Advanced SCLC, LCNEC	Standard of care, slows disease progression
	Carboplatin	Alternative for cisplatin-intolerant patients	Less efficacy data compared to cisplatin
	Adjuvant Chemotherapy (EP)	Stage IB-IIIA LCNEC	Improves post-resection survival
	Radiotherapy	Advanced inoperable NETs	Local disease control
	Somatostatin Analogs (Octreotide, Lanreotide)	Well-differentiated carcinoids	Reduces tumor growth
	Radioembolization with Labeled Somatostatin Analogs	Advanced NETs not eligible for surgery	Controls progression and improves quality of life
PanNETs	Somatostatin analogs (Octreotide, Lanreotide)	Well-differentiated functional and nonfunctional PanNETs	Symptom relief and tumor growth control
	Everolimus (mTOR inhibitor)	Advanced PanNETs	Prolongs progression-free survival
	Sunitinib (Tyrosine Kinase Inhibitor)	Advanced PanNETs	Inhibits tumor angiogenesis and growth
	PRRT with Lutetium-177	Metastatic PanNETs expressing SSTR	Effective tumor growth control
	Chemotherapy (Streptozocin, Temozolomide, Capecitabine)	High-grade PanNETs	Cytotoxic effect on rapidly proliferating cells
	Epigenetic Therapy (DNMT and HDAC inhibitors)	Emerging therapy for PanNETs	Restores tumor suppressor gene expression
MTC	Total Thyroidectomy	First-line treatment	Complete removal of the thyroid gland, reducing tumor burden
	Lymph Node Dissection	Regional metastasis	Removal of affected lymph nodes, improving local control
	TKIs	Advanced/metastatic MTC	Inhibits RET and other kinases, slowing tumor progression
	Selective RET Inhibitors (e.g., Selpercatinib, Pralsetinib)	RET-mutated MTC	Blocks RET-driven tumor growth, potentially improving survival
	Peptide Receptor Radionuclide Therapy (PRRT)	Somatostatin receptor-positive MTC	Delivers targeted radiation to tumor cells, reducing tumor size
	Chemotherapy	Refractory MTC cases	Cytotoxic drug-induced tumor cell death, palliative effect

## Data Availability

Not applicable.

## References

[1] Fraenkel M., Kim M., Faggiano A., De Herder W.W., Valk G.D., Knowledge NETwork (2014). Incidence of gastroenteropancreatic neuroendocrine tumours: A systematic review of the literature. Endocr. Relat. Cancer.

[2] Sultana Q., Kar J., Verma A., Sanghvi S., Kaka N., Patel N., Sethi Y., Chopra H., Kamal M.A., Greig N.H. (2023). A Comprehensive Review on Neuroendocrine Neoplasms: Presentation, Pathophysiology and Management. J. Clin. Med..

[3] Colao A., de Nigris F., Modica R., Napoli C. (2020). Clinical Epigenetics of Neuroendocrine Tumors: The Road Ahead. Front. Endocrinol..

[4] Man D., Wu J., Shen Z., Zhu X. (2018). Prognosis of patients with neuroendocrine tumor: A SEER database analysis. Cancer Manag. Res..

[5] Yao J.C., Hassan M.M., Phan A.T., Dagohoy C.G., Leary C.C., Mares J.E., Abdalla E.K., Fleming J.B., Vauthey J.-N., Rashid A. (2008). One Hundred Years After “Carcinoid”: Epidemiology of and Prognostic Factors for Neuroendocrine Tumors in 35,825 Cases in the United States. J. Clin. Oncol..

[6] Dreijerink K.M., Hackeng W.M., Singhi A.D., Heaphy C.M., Brosens L.A. (2022). Clinical implications of cell-of-origin epigenetic characteristics in non-functional pancreatic neuroendocrine tumors. J. Pathol..

[7] Webster A.P., Thirlwell C. (2024). The Molecular Biology of Midgut Neuroendocrine Neoplasms. Endocr. Rev..

[8] Moore L.D., Le T., Fan G. (2013). DNA methylation and its basic function. Neuropsychopharmacology.

[9] Shenker N., Flanagan J.M. (2012). Intragenic DNA methylation: Implications of this epigenetic mechanism for cancer research. Br. J. Cancer.

[10] Esteller M. (2008). Epigenetics in Cancer. N. Engl. J. Med..

[11] Chan A.O.-O., Kim S.G., Bedeir A., Issa J.-P., Hamilton S.R., Rashid A. (2003). CpG island methylation in carcinoid and pancreatic endocrine tumors. Oncogene.

[12] Verdugo A.D., Crona J., Starker L., Stålberg P., Åkerström G., Westin G., Hellman P., Björklund P. (2014). Global DNA methylation patterns through an array-based approach in small intestinal neuroendocrine tumors. Endocr. Relat. Cancer.

[13] Lawrence B., Gustafsson B.I., Kidd M., Pavel M., Svejda B., Modlin I.M. (2011). The clinical relevance of chromogranin A as a biomarker for gastroenteropancreatic neuroendocrine tumors. Endocrinol. Metab. Clin. N. Am..

[14] Zatelli M.C., Torta M., Leon A., Ambrosio M.R., Gion M., Tomassetti P., De Braud F., Fave G.D., Dogliotti L., degli Uberti E.C. (2007). Chromogranin A as a marker of neuroendocrine neoplasia: An Italian Multicenter Study. Endocr. Relat. Cancer.

[15] Kidd M., Bodei L., Modlin I.M. (2016). Chromogranin A: Any relevance in neuroendocrine tumors?. Curr. Opin. Endocrinol. Diabetes.

[16] Isgrò M.A., Bottoni P., Scatena R. (2015). Neuron-Specific Enolase as a Biomarker: Biochemical and Clinical Aspects. Adv. Exp. Med. Biol..

[17] Baudin E., Gigliotti A., Ducreux M., Ropers J., Comoy E., Sabourin J., Bidart J., Cailleux A., Bonacci R., Ruffié P. (1998). Neuron-specific enolase and chromogranin A as markers of neuroendocrine tumours. Br. J. Cancer.

[18] Wang C., Wu N., Pei B., Ma X., Yang W. (2023). Claudin and pancreatic cancer. Front. Oncol..

[19] Liu L., Broaddus R.R., Yao J.C., Xie S., A White J., Wu T.-T., Hamilton S.R., Rashid A. (2005). Epigenetic alterations in neuroendocrine tumors: Methylation of RAS-association domain family 1, isoform A and p16 genes are associated with metastasis. Mod. Pathol..

[20] Miura K., Shimizu K., Ide S., Mishima S., Matsuoka S., Takeda T., Eguchi T., Hamanaka K., Uehara T. (2021). A Novel Strategy for the Diagnosis of Pulmonary High-Grade Neuroendocrine Tumor. Diagnostics.

[21] Sen T., Dotsu Y., Corbett V., Puri S., Sen U.S., Boyle T.A., Mack P., Hirsch F., Aljumailyet R., Naqash A.R. (2025). Pulmonary neuroendocrine neoplasms: The molecular landscape, therapeutic challenges, and diagnosis and management strategies. Lancet Oncol..

[22] Sun T.Y., Hendifar A., Padda S.K. (2022). Lung Neuroendocrine Tumors: How Does Molecular Profiling Help?. Curr. Oncol. Rep..

[23] Du C., Tan L., Xiao X., Xin B., Xiong H., Zhang Y., Ke Z., Yin J. (2024). Detection of the DNA methylation of seven genes contribute to the early diagnosis of lung cancer. J. Cancer Res. Clin. Oncol..

[24] Pfeifer G.P., Kernstine K.H. (2017). DNA methylation biomarkers in lung cancer diagnosis: Closer to practical use?. Transl. Cancer Res..

[25] Warton K., Mahon K.L., Samimi G. (2016). Methylated circulating tumor DNA in blood: Power in cancer prognosis and response. Endocr. Relat. Cancer.

[26] Balgkouranidou I., Chimonidou M., Milaki G., Tsaroucha E., Kakolyris S., Georgoulias V., Lianidou E. (2016). SOX17 promoter methylation in plasma circulating tumor DNA of patients with non-small cell lung cancer. Clin. Chem. Lab. Med..

[27] Li D.-M., Li G.-S., Li J.-D., Chen F., Huang H., Huang W.-Y., Huang Z.-G., Dang Y.-W., Tang Y.-L., Tang Z.-Q. (2024). Clinical significance and prospective mechanism of increased CDKN2A expression in small cell lung cancer. Clin. Transl. Oncol..

[28] Fisseler-Eckhoff A., Demes M. (2012). Neuroendocrine Tumors of the Lung. Cancers.

[29] Tests for Lung Carcinoid Tumors|American Cancer Society. https://www.cancer.org/cancer/types/lung-carcinoid-tumor/detection-diagnosis-staging/how-diagnosed.html.

[30] Derks J.L., Dingemans A.C., van Suylen R., Bakker M.A.D., Damhuis R.A.M., Broek E.C.v.D., Speel E., Thunnissen E. (2019). Is the sum of positive neuroendocrine immunohistochemical stains useful for diagnosis of large cell neuroendocrine carcinoma (LCNEC) on biopsy specimens?. Histopathology.

[31] Bakker M.A.D., Willemsen S., Grünberg K., Noorduijn L.A., Van Oosterhout M.F.M., Van Suylen R.J., Timens W., Vrugt B., Tilburg A.W., Thunnissen F.B.J.M. (2010). Small cell carcinoma of the lung and large cell neuroendocrine carcinoma interobserver variability. Histopathology.

[32] Thunnissen E., Borczuk A.C., Flieder D.B., Witte B., Beasley M.B., Chung J.-H., Dacic S., Lantuejoul S., Russell P.A., den Bakker M. (2017). The Use of Immunohistochemistry Improves the Diagnosis of Small Cell Lung Cancer and Its Differential Diagnosis. An International Reproducibility Study in a Demanding Set of Cases. J. Thorac. Oncol..

[33] Kujtan L., Muthukumar V., Kennedy K.F., Davis J.R., Masood A., Subramanian J. (2018). The Role of Systemic Therapy in the Management of Stage I Large Cell Neuroendocrine Carcinoma of the Lung. J. Thorac. Oncol..

[34] Le Treut J., Sault M.C., Lena H., Souquet P.J., Vergnenegre A., Le Caer H., Berard H., Boffa S., Monnet I., Damotte D. (2013). Multicentre phase II study of cisplatin-etoposide chemotherapy for advanced large-cell neuroendocrine lung carcinoma: The GFPC 0302 study. Ann. Oncol..

[35] Ramirez R.A., Thomas K., Jacob A., Lin K., Bren-Mattison Y., Chauhan A. (2021). Adjuvant therapy for lung neuroendocrine neoplasms. World J. Clin. Oncol..

[36] Raman V., Jawitz O.K., Yang C.-F.J., Tong B.C., D’amico T.A., Berry M.F., Harpole D.H. (2019). Adjuvant Therapy for Patients with Early Large Cell Lung Neuroendocrine Cancer: A National Analysis. Ann. Thorac. Surg..

[37] Zhu S., Wang X., Li H., Zhao P., Liu J., Zhang L., Cheng Y. (2024). Advances in genetic profile and therapeutic strategy of pulmonary large cell neuroendocrine carcinoma. Front. Med..

[38] Andrini E., Marchese P.V., De Biase D., Mosconi C., Siepe G., Panzuto F., Ardizzoni A., Campana D., Lamberti G. (2022). Large Cell Neuroendocrine Carcinoma of the Lung: Current Understanding and Challenges. J. Clin. Med..

[39] Kang K., Li B., Wang S., Wang J., Liang X. (2024). Clinical characteristics and treatment management of combined large cell neuroendocrine carcinoma, a subtype of large cell neuroendocrine carcinoma. Front. Oncol..

[40] Al-Toubah T., Montilla-Soler J., El-Haddad G., Haider M., Strosberg J. (2023). Somatostatin Receptor Expression in Lung Neuroendocrine Tumors: An Analysis of DOTATATE PET Scans. J. Nucl. Med..

[41] Baladi A., Tafenzi H.A., Zouiten O., Afani L., Essaadi I., El Fadli M., Belbaraka R. (2025). Immunotherapy for Elderly Patients with Advanced Non-Small Cell Lung Cancer: Challenges and Perspectives. Int. J. Mol. Sci..

[42] Roussot N., Kaderbhai C., Ghiringhelli F. (2025). Targeting Immune Checkpoint Inhibitors for Non-Small-Cell Lung Cancer: Beyond PD-1/PD-L1 Monoclonal Antibodies. Cancers.

[43] Ruggiero R., Balzano N., Di Napoli R., Fraenza F., Pentella C., Riccardi C., Donniacuo M., Tesorone M., Danesi R., Del Re M. (2023). Do peripheral neuropathies differ among immune checkpoint inhibitors? Reports from the European post-marketing surveillance database in the past 10 years. Front. Immunol..

[44] Rudin C.M., Reck M., Johnson M.L., Blackhall F., Hann C.L., Yang J.C.-H., Bailis J.M., Bebb G., Goldrick A., Umejiego J. (2023). Emerging therapies targeting the delta-like ligand 3 (DLL3) in small cell lung cancer. J. Hematol. Oncol..

[45] Chan J., Kulke M. (2014). Targeting the mTOR signaling pathway in neuroendocrine tumors. Curr. Treat. Options Oncol..

[46] Exner S., Arrey G., Prasad V., Grötzinger C. (2021). mTOR Inhibitors as Radiosensitizers in Neuroendocrine Neoplasms. Front. Oncol..

[47] Tanimura K., Yamada T., Omura A., Shiotsu S., Kataoka N., Takeda T., Taniguchi R., Yamada T., Takeuchi M., Chihara Y. (2021). The Impact of VEGF Inhibition on Clinical Outcomes in Patients With Advanced Non-Small Cell Lung Cancer Treated With Immunotherapy: A Retrospective Cohort Study. Front. Oncol..

[48] Sportiello L., Di Napoli R., Balzano N., Mascolo A., Ruggiero R., Di Costanzo L., Monaco D., Maniscalco G.T., Capuano A. (2023). Disease-Modifying Therapies (DMTs) in Pregnant and Lactating Women with Multiple Sclerosis: Analysis of Real-World Data from EudraVigilance Database. Pharmaceuticals.

[49] Yin F., Wu Z.-H., Lai J.-P. (2022). New insights in diagnosis and treatment of gastroenteropancreatic neuroendocrine neoplasms. World J. Gastroenterol..

[50] Nagtegaal I.D., Odze R.D., Klimstra D., Paradis V., Rugge M., Schirmacher P., Washington K.M., Carneiro F., Cree I.A., The WHO Classification of Tumours Editorial Board (2020). The 2019 WHO classification of tumours of the digestive system. Histopathology.

[51] Metz D.C., Jensen R.T. (2008). Gastrointestinal neuroendocrine tumors: Pancreatic endocrine tumors. Gastroenterology.

[52] Yan J., Yu S., Jia C., Li M., Chen J. (2020). Molecular subtyping in pancreatic neuroendocrine neoplasms: New insights into clinical, pathological unmet needs and challenges. Biochim. Biophys. Acta Rev. Cancer.

[53] Halfdanarson T.R., Rabe K.G., Rubin J., Petersen G.M. (2008). Pancreatic neuroendocrine tumors (PNETs): Incidence, prognosis and recent trend toward improved survival. Ann. Oncol..

[54] Halfdanarson T.R., Rubin J., Farnell M.B., Grant C.S., Petersen G.M. (2008). Pancreatic endocrine neoplasms: Epidemiology and prognosis of pancreatic endocrine tumors. Endocr. Relat. Cancer.

[55] Luce A., Lombardi A., Ferri C., Zappavigna S., Tathode M.S., Miles A.K., Boocock D.J., Vadakekolathu J., Bocchetti M., Alfano R. (2022). A Proteomic Approach Reveals That miR-423-5p Modulates Glucidic and Amino Acid Metabolism in Prostate Cancer Cells. Int. J. Mol. Sci..

[56] Crabtree J.S. (2022). Epigenetic Regulation in Gastroenteropancreatic Neuroendocrine Tumors. Front. Oncol..

[57] Bevere M., Gkountakos A., Martelli F.M., Scarpa A., Luchini C., Simbolo M. (2023). An Insight on Functioning Pancreatic Neuroendocrine Neoplasms. Biomedicines.

[58] Marini F., Giusti F., Tonelli F., Brandi M.L. (2021). Pancreatic Neuroendocrine Neoplasms in Multiple Endocrine Neoplasia Type 1. Int. J. Mol. Sci..

[59] Wang F., Xu X., Ye Z., Qin Y., Yu X., Ji S. (2021). Prognostic Significance of Altered ATRX/DAXX Gene in Pancreatic Neuroendocrine Tumors: A Meta-Analysis. Front. Endocrinol..

[60] Ciobanu O.A., Martin S.C., Herlea V., Fica S. (2022). Insights into Epigenetic Changes Related to Genetic Variants and Cells-of-Origin of Pancreatic Neuroendocrine Tumors: An Algorithm for Practical Workup. Cancers.

[61] Tirosh A., Kebebew E. (2020). Genetic and epigenetic alterations in pancreatic neuroendocrine tumors. J. Gastrointest. Oncol..

[62] Niina Y., Fujimori N., Nakamura T., Igarashi H., Oono T., Nakamura K., Kato M., Jensen R.T., Ito T., Takayanagi R. (2012). The current strategy for managing pancreatic neuroendocrine tumors in multiple endocrine neoplasia type 1. Gut Liver.

[63] Romero-Garcia S., Prado-Garcia H., Carlos-Reyes A. (2020). Role of DNA Methylation in the Resistance to Therapy in Solid Tumors. Front. Oncol..

[64] Gao J., Shi W., Wang J., Guan C., Dong Q., Sheng J., Zou X., Xu Z., Ge Y., Yang C. (2024). Research progress and applications of epigenetic biomarkers in cancer. Front. Pharmacol..

[65] Kaz A.M., Grady W.M. (2014). Epigenetic biomarkers in esophageal cancer. Cancer Lett..

[66] Yamada Y., Venkadakrishnan V.B., Mizuno K., Bakht M., Ku S.-Y., Garcia M.M., Beltran H. (2023). Targeting DNA methylation and B7-H3 in RB1-deficient and neuroendocrine prostate cancer. Sci. Transl. Med..

[67] Marks D.L., Olson R.L., E Fernandez-Zapico M. (2016). Epigenetic control of the tumor microenvironment. Epigenomics.

[68] Cuthbertson D.J., Barriuso J., Lamarca A., Manoharan P., Westwood T., Jaffa M., Fenwick S.W., Nuttall C., Lalloo F., Prachalias A. (2021). The Impact of 68Gallium DOTA PET/CT in Managing Patients With Sporadic and Familial Pancreatic Neuroendocrine Tumours. Front. Endocrinol..

[69] Abi-Raad R., Lavik J.-P., Barbieri A.L., Zhang X., Adeniran A.J., Cai G. (2019). Grading Pancreatic Neuroendocrine Tumors by Ki-67 Index Evaluated on Fine-Needle Aspiration Cell Block Material. Am. J. Clin. Pathol..

[70] Thiis-Evensen E., Kjellman M., Knigge U., Gronbaek H., Schalin-Jäntti C., Welin S., Sorbye H., Schneider M.d.P., Belusa R., The Nordic NET Biomarker Group (2022). Plasma protein biomarkers for the detection of pancreatic neuroendocrine tumors and differentiation from small intestinal neuroendocrine tumors. J. Neuroendocr..

[71] Sharma J., Duque M., Saif M.W. (2013). Emerging therapies and latest development in the treatment of unresectable pancreatic neuroendocrine tumors: An update for clinicians. Ther. Adv. Gastroenterol..

[72] Giri S., Sahoo J. (2024). Advancements in medical treatment for pancreatic neuroendocrine tumors: A beacon of hope. World J. Gastroenterol..

[73] Chan J.A., Stuart K., Earle C.C., Clark J.W., Bhargava P., Miksad R., Blaszkowsky L., Enzinger P.C., Meyerhardt J.A., Zheng H. (2012). Prospective study of bevacizumab plus temozolomide in patients with advanced neuroendocrine tumors. J. Clin. Oncol..

[74] Neychev V., Steinberg S.M., Cottle-Delisle C., Merkel R., Nilubol N., Yao J., Meltzer P., Pacak K., Marx S., Kebebew E. (2015). Mutation-targeted therapy with sunitinib or everolimus in patients with advanced low-grade or intermediate-grade neuroendocrine tumours of the gastrointestinal tract and pancreas with or without cytoreductive surgery: Protocol for a phase II clinical trial. BMJ Open.

[75] Partelli S., Bertani E., Bartolomei M., Perali C., Muffatti F., Grana C.M., Lena M.S., Doglioni C., Crippa S., Fazio N. (2018). Peptide receptor radionuclide therapy as neoadjuvant therapy for resectable or potentially resectable pancreatic neuroendocrine neoplasms. Surgery.

[76] Fernández-Ferreira R., De la Peña-López I.R., Zamudio-Coronado K.W., Delgado-Soler L.A., Torres-Pérez M.E., Ríos C.B.-D.L., Cortés-González R. (2021). Calcitonin-Negative Neuroendocrine Carcinoma of the Thyroid Gland: Case Report and Literature Review. Case Rep. Oncol..

[77] Johansson E., Andersson L., Örnros J., Carlsson T., Ingeson-Carlsson C., Liang S., Dahlberg J., Jansson S., Parrillo L., Zoppoli P. (2015). Revising the embryonic origin of thyroid C cells in mice and humans. Development.

[78] Chiba T. (2024). Molecular Pathology of Thyroid Tumors: Essential Points to Comprehend Regarding the Latest WHO Classification. Biomedicines.

[79] Raue F., Kotzerke J., Reinwein D., Deckart H., Ritter M., Seif F., Buhr H., Beyer J., Schober O., Becker W. (1993). Prognostic factors in medullary thyroid carcinoma: Evaluation of 741 patients from the German Medullary Thyroid Carcinoma Register. Clin. Investig..

[80] Shen C., Shi X., Wen D., Zhang Y., Du Y., Zhang Y., Ma B., Tang H., Yin M., Huang N. (2024). Comprehensive DNA Methylation Profiling of Medullary Thyroid Carcinoma: Molecular Classification, Potential Therapeutic Target, and Classifier System. Clin. Cancer Res..

[81] Romei C., Ciampi R., Casella F., Tacito A., Torregrossa L., Ugolini C., Basolo F., Materazzi G., Vitti P., Elisei R. (2018). RET mutation heterogeneity in primary advanced medullary thyroid cancers and their metastases. Oncotarget.

[82] Chang Y.-S., Chang C.-C., Huang H.-Y., Lin C.-Y., Yeh K.-T., Chang J.-G. (2018). Detection of Molecular Alterations in Taiwanese Patients with Medullary Thyroid Cancer Using Whole-Exome Sequencing. Endocr. Pathol..

[83] Ciampi R., Romei C., Ramone T., Prete A., Tacito A., Cappagli V., Bottici V., Viola D., Torregrossa L., Ugolini C. (2019). Genetic Landscape of Somatic Mutations in a Large Cohort of Sporadic Medullary Thyroid Carcinomas Studied by Next-Generation Targeted Sequencing. iScience.

[84] Citarella A., Besharat Z.M., Trocchianesi S., Autilio T.M., Verrienti A., Catanzaro G., Splendiani E., Spinello Z., Cantara S., Zavattari P. (2023). Circulating cell-free DNA (cfDNA) in patients with medullary thyroid carcinoma is characterized by specific fragmentation and methylation changes with diagnostic value. Biomark. Res..

[85] Daumerie C., Maiter D., Gruson D. (2013). Serum calcitonin estimation in medullary thyroid cancer: Basal or stimulated levels?. Thyroid. Res..

[86] Censi S., Manso J., Mian C. (2023). Other markers of medullary thyroid cancer, not only calcitonin. Eur. J. Endocrinol..

[87] Della Monica R., Cuomo M., Visconti R., di Mauro A., Buonaiuto M., Costabile D., De Riso G., Di Risi T., Guadagno E., Tafuto R. (2022). Evaluation of MGMT Gene Methylation in Neuroendocrine Neoplasms. Oncol. Res. Featur. Preclin. Clin. Cancer Ther..

[88] Liu S.-C. (2024). Circulating tumor DNA in liquid biopsy: Current diagnostic limitation. World J. Gastroenterol..

[89] Allelein S., Ehlers M., Morneau C., Schwartz K., Goretzki P.E., Seppel T., Feldkamp J., Krieg A., Knoefel W.T., Kuebart A. (2018). Measurement of Basal Serum Calcitonin for the Diagnosis of Medullary Thyroid Cancer. Horm. Metab. Res..

[90] Lee T., Teng T.Z.J., Shelat V.G. (2020). Carbohydrate antigen 19-9-tumor marker: Past, present, and future. World J. Gastrointest. Surg..

[91] Trimboli P., Lauretta R., Barnabei A., Valabrega S., Romanelli F., Giovanella L., Appetecchia M. (2018). Procalcitonin as a postoperative marker in the follow-up of patients affected by medullary thyroid carcinoma. Int. J. Biol. Markers.

[92] Trimboli P., Seregni E., Treglia G., Alevizaki M., Giovanella L. (2015). Procalcitonin for detecting medullary thyroid carcinoma: A systematic review. Endocr. Relat. Cancer.

[93] de Andrade F.A., Bulzico D., Corbo R., Vaisman F. (2024). Is peptide receptor radionuclide therapy still a promising option for medullary thyroid carcinoma?. Endocrine.

[94] Lind P., Jacobson A., Nordenström E., Johansson L., Wallin G., Daskalakis K. (2024). Diagnostic sensitivity of fine-needle aspiration cytology in thyroid cancer. Sci. Rep..

[95] Tomita T. (2021). Significance of chromogranin A and synaptophysin in medullary thyroid carcinoma. Bosn. J. Basic Med. Sci..

[96] Clayman G.L., El-Baradie T.S. (2003). Medullary thyroid cancer. Otolaryngol. Clin. N. Am..

[97] Martins-Costa M.C., Cunha L.L., Lindsey S.C., Camacho C.P., Dotto R.P., Furuzawa G.K., A Sousa M.S., Kasamatsu T.S., Kunii I.S., Martins M.M. (2016). M918V RET mutation causes familial medullary thyroid carcinoma: Study of 8 affected kindreds. Endocr. Relat. Cancer.

[98] Machens A., Lorenz K., Brandenburg T., Führer D., Weber F., Dralle H. (2024). Latest Progress in Risk-Adapted Surgery for Medullary Thyroid Cancer. Cancers.

[99] Kim B.H., Kim I.J. (2016). Recent Updates on the Management of Medullary Thyroid Carcinoma. Endocrinol. Metab..

[100] Efstathiadou Z.A., Tsentidis C., Bargiota A., Daraki V., Kotsa K., Ntali G., Papanastasiou L., Tigas S., Toulis K., Pazaitou-Panayiotou K. (2021). Benefits and Limitations of TKIs in Patients with Medullary Thyroid Cancer: A Systematic Review and Meta-Analysis. Eur. Thyroid J..

[101] Ringel M.D. (2021). New Horizons: Emerging Therapies and Targets in Thyroid Cancer. J. Clin. Endocrinol. Metab..

[102] Grewal R.K., Ho A., Schöder H. (2016). Novel Approaches to Thyroid Cancer Treatment and Response Assessment. Semin. Nucl. Med..

